# Noncoding RNAs as regulators of FOSL1 in cancer

**DOI:** 10.3389/fimmu.2025.1599674

**Published:** 2025-08-01

**Authors:** Xiaochang Wang, Li Wang, Shoushi Wang, Jinjie Zhang, Xueyang Wang, Ting Zhang, Linwei Li, Jin Wei, Yi Zhao, Zhixia Zhou

**Affiliations:** ^1^ Institute for Translational Medicine, The Affiliated Hospital of Qingdao University, Qingdao Medical College, Qingdao University, Qingdao, China; ^2^ Department of Anesthesia and Perioperative Medicine, Qingdao Central Hospital, University of Health and Rehabilitation Sciences, Qingdao, Shandong, China

**Keywords:** FOSL1, ncRNA, cancer, immune escape, targeted therapy

## Abstract

The AP-1 transcription factor FOSL1, also known as Fra-1, is a crucial oncoprotein that plays an important role in human tumor progression and metastasis and has thus emerged as a promising therapeutic target. FOSL1 regulates the expression of a large protein-coding gene network, and this molecular mechanism can promote the progression of tumors. Interestingly, recent studies have shown that FOSL1 can also achieve the same protumor effect by regulating certain noncoding RNAs (ncRNAs). However, more studies have shown that ncRNAs can regulate the expression and activity of FOSL1, thereby affecting the occurrence and development of tumors, which indicates that ncRNAs can be regulators of FOSL1 in cancer. In this review, we first provide a comprehensive overview of the expression and function of FOSL1 and ncRNAs in tumors and then focus on the mutual regulatory relationship between ncRNAs and FOSL1, as well as their regulatory effects on and mechanisms of tumor progression. In addition, we further explored the potential clinical applications of the FOSL1-ncRNA system in cancer treatment, providing a theoretical basis for the study of FOSL1 and/or ncRNA-related molecular markers or targeted therapies.

## 1 Introduction

The study of activator protein-1 (AP-1), which can be purified from HeLa cells by sequence-specific DNA affinity chromatography, began in 1980’s (1). First, AP-1 was reported to bind to TPA (tissue plasminogen activator) response elements (TREs) located in the promoter region of some TPA-induced genes, such as matrix lysin and collagenase, and promote the transcription of these genes (2). Subsequent studies have revealed that AP-1 has a wide range of biological functions, such as being a transcription factor involved in cell survival, apoptosis, migration, immune escape, cell transformation and metastasis (3), and participates in the process of hematopoietic differentiation (4), angiogenesis, inflammatory reactions and carcinogenesis (3).This wide range of functions carried out by AP-1 is inseparable from its structural composition. AP-1 is a homodimeric or heterodimeric protein composed of Jun family proteins (c-Jun, JunB, and JunD), Fos family proteins (c-Fos, FosB, FOSL1/Fra-1 [Fos-related antigen 1], and FOSL2/Fra-2), activating transcription factor (ATF), and muscle aponeurotic fibrosarcoma (MAF) family proteins, which binds to DNA through the basic leucine zipper (bZIP) structure (5). Different subunit compositions result in different affinities between AP-1 and different binding sequences, leading to different biological activities with different target genes (6). In further research on the structure and function of AP-1, the Fos family has attracted the attention of researchers, especially FOSL1, as it differs from c-Fos and c-Jun in that it does not have a transactivation function.

FOSL1 was discovered in the last century, and its gene product has extensive amino acid homology and similar antigenicity to the c-Fos protein (7). Early research on FOSL1 coincided mainly with research on AP-1 and c-Fos. FOSL1 can be induced by serum in fibroblasts and is an early gene that participates in the cell’s response to environmental stimuli. However, during serum induction, the expression of FOSL1 is upregulated later than the expression of c-Fos, suggesting that FOSL1 can be positively regulated by AP-1 (8), which is unique in the Fos family. In addition, FOSL1 can negatively regulate the activity of AP-1 in circulating cells and stabilize AP-1 by binding to Jun and subsequently increasing its binding to DNA (7). FOSL1 is expressed and plays a role in epidermal tissue, the nervous system, and bone (9), but its constitutive levels are not high in other normal tissues, and there is no significant tissue specificity. Furthermore, FOSL1 is expressed mainly in the nuclei of proliferating cells and is related to the synthesis of genetic material during the cell cycle (10), which has a significant effect on the progression of the cell cycle. Therefore, FOSL1 was subsequently confirmed to be related to cellular aging, such as playing a key role in angiotensin-induced vascular aging (11), while reducing cell proliferation and inducing apoptosis on the basis of the cellular environment. Research has revealed that FOSL1, which is often highly expressed in tumor tissues and has an effect on malignant transformation, proliferation, invasion, anti-apoptosis and drug resistance of tumor cells, is related to tumor progression (12). Moreover, as the important role of FOSL1 in cancer progression has been confirmed, its regulatory mechanism, which involves many tumor-related genes and signaling pathways and even noncoding genes, such as noncoding RNAs (ncRNAs), has also attracted attention.

ncRNA refers to RNA that is transcribed from the genome and does not encode protein. According to their length, shape and location, ncRNAs can be divided into at least four types: long noncoding RNAs (lncRNAs), microRNAs (miRNAs), circular RNAs (circRNAs) and Piwi-interacting RNAs (piRNAs). Although they cannot or rarely encode proteins, they can interact with other encoding RNAs, DNA, or proteins to regulate gene expression at the epigenetic, transcriptional, and posttranslational levels (13). Therefore, ncRNAs are involved in a wide range of complex physiological and pathological processes. Research on many types of cancer has revealed that many ncRNAs can promote or inhibit the development of tumors. They play important regulatory roles in various tumor activities, such as proliferation, apoptosis, invasion, metastasis, immune escape, and the therapeutic response of tumor cells (14). Currently, many preclinical and clinical trials have been initiated to confirm the potential value of ncRNAs as biomarkers for tumor diagnosis or prognosis (15). Moreover, the development of anticancer drugs based on ncRNAs has gained significant momentum and may lead to breakthroughs. Interestingly, recent studies have shown that there is a complex regulatory relationship between ncRNAs and FOSL1, which can significantly influence the occurrence, development and prognosis of cancer by working together with multiple molecules and forming a complex signaling network (16).

This type of mutual regulation is not only found between ncRNAs and FOSL1. Other members of the Fos superfamily can interact with different ncRNAs to participate in the development of cancer. For example, c-Fos, an oncogene located at 14q24.3, can form a heterodimer with c-Jun that can promote cancer. In non-small cell lung carcinoma, c-Fos/c-Jun upregulate cyclin D1 through IL-7 to improve the invasion and proliferation of tumors (17). In gastric cancer, RPRD1B can adjust the intake and synthesis of fatty acids through the upregulation of c-Fos/c-Jun, and c-Fos/c-Jun can also decrease the degradation of RPRD1B by increasing the content of the lncRNA NEAT (18). The function of FosB in the progression of cancer is more interesting. In glioma, Fosb promotes the expression of cleaved caspase-3 and then inhibits the apoptosis of tumor cells (19). FosB can also play a dual role in the development of lung cancer depending on the phenotype of p53 (20). LINC00963 can recruit Fosb to the ubiquitin protein ligase E3C (UBE3C) promoter and increase the radioresistance of breast cancer through UBE3C (21). Moreover, Fra-2 also has the closest relationship with ncRNAs in tumor biology. In non-small cell lung cancer, the lncRNA ITGB2-AS1 can directly bind to the Fra-2/NAMPT axis and then inhibit ferroptosis induced by p53, which promotes cisplatin resistance (22). Additionally, miR-301a inhibits the Fra-2/GLIPR1 (GLI pathogenesis-related 1) axis to increase cisplatin resistance in lung cancer (23). In pancreatic ductal adenocarcinoma (PDAC), the abnormal downregulation of miR-15a leads to the upregulation of Fra-2, which can then mediate the adaptation of tumor cells to nutrient deprivation via the Fra-2/IGF1R (IGF1 receptor) axis (24). These data indicate that the Fos-ncRNA axis plays an important regulatory role in tumor biology and suggest the clinical potential of tumor therapy targeting this axis.

In this review, we first provide an overview of the basic information concerning FOSL1 and ncRNAs, as well as their regulatory roles in tumors. We focused on the mutual relationship and potential molecular mechanisms between ncRNAs and FOSL1 during cancer development. In addition, we further explored the potential clinical application of the FOSL1-ncRNA system in cancer treatment, which provides a theoretical basis for research on molecular markers or targeted therapies related to FOSL1 and/or ncRNA.

## 2 FOSL1 in tumors

**Figure 1:**
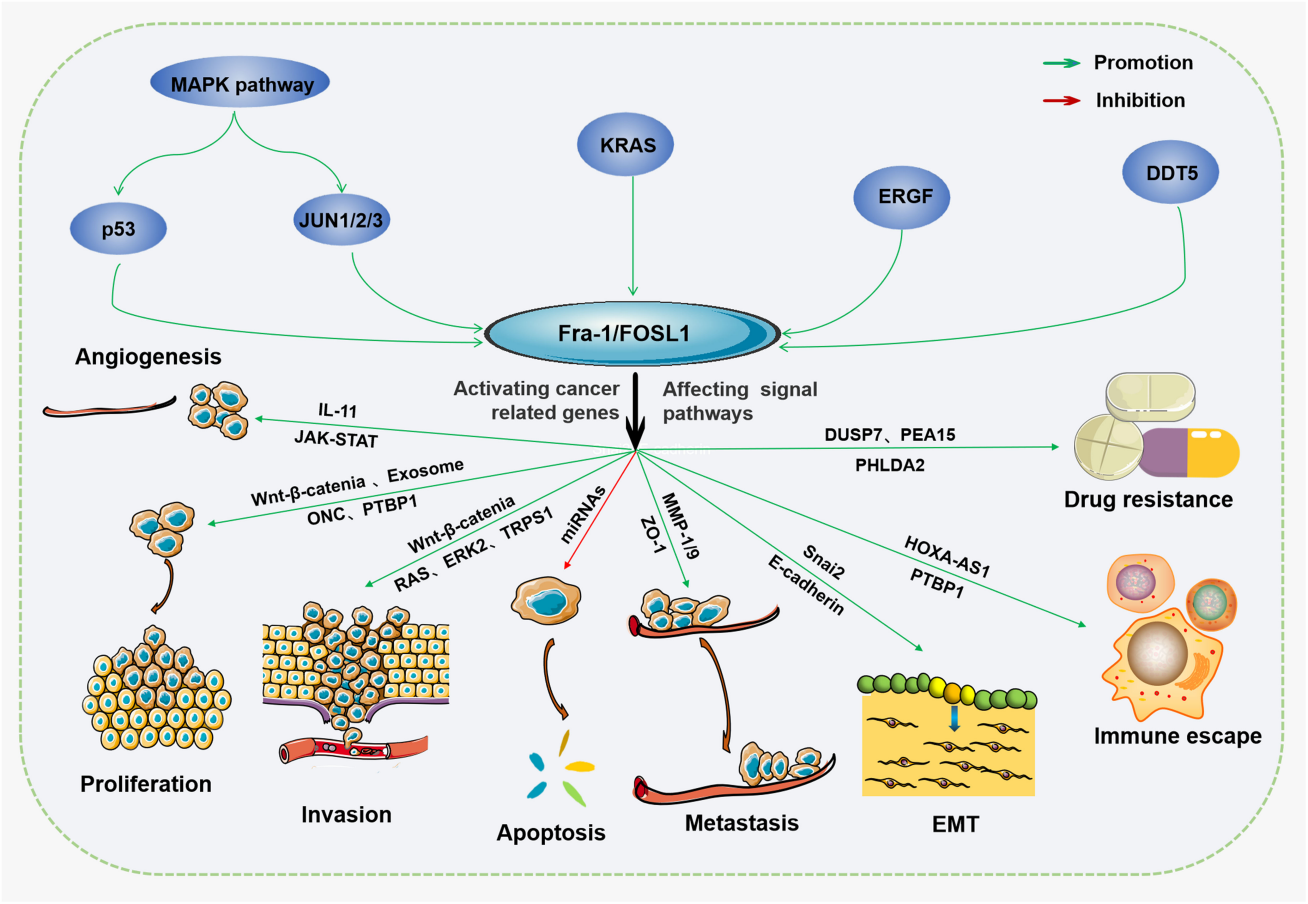
The protumor effects of FOSL1 Fra-1/FOSL1 can promote tumor growth, proliferation, and metastasis, EMT, immune escape, and drug resistance and inhibit apoptosis by activating or inhibiting cancer-related genes or affecting tumor-associated signal transduction pathways.

FOSL1 is a 271-amino acid protein encoded by the *FOSL1* gene located at 11q.13.1. The expression of FOSL1 is abnormal in many types of tumors, and it is upregulated in many tumors; however, its functions and content are not the same in different tumor types (25). FOSL1 affects the progression of cancer mainly through the transcription of a series of tumor-related genes by binding the promoters, introns and distal enhancers in the binding regulatory sequence (26, 27). In different tumors, the target of FOSL1 is specific; for example, in gastric cancer, FOSL1 can promote the proliferation of tumor cells through the RAS-ERK (extracellular signal-regulated kinase) and PI3K (phosphatidylinositol 3-kinase)-AKT pathways. However, in thyroid cancer, cell division can be promoted by the ability of FOSL1 to regulate the cell cycle (12). Overall, FOSL1 has a strong influence on tumor progression, including promoting proliferation, metastasis, invasion, antiapoptosis activity, and drug resistance and increasing tumor heterogeneity (28), as shown in **Figure 1**. Moreover, FOSL1 is involved in other important signaling pathways that affect tumor progression, such as the interleukin-6/signal transducer and activator of transcription 3 (IL-6/Stat3) pathway (29), the Wnt-β-catenin pathway (30), and other major carcinogenic pathways. For example, FOSL1 can induced the formation of superenhancers (SEs) that bind to tumor-related genes (such as metastasis-promoting genes), thus promoting gene expression and cancer cell migration (31). Tumors may contain multiple types of clones at the same time, among which tumor stem cells (CSCs) proliferate rapidly and have strong invasiveness, which is considered one of the reasons why tumors easily recur and are difficult to treat (32). FOSL1 can also promote the reprogramming of cancer cells with other phenotypes of CSCs, thus promoting tumor metastasis (33). Moreover, FOSL1 can also induce phenotypic changes in CSCs, increasing the heterogeneity of tumors and promoting their development toward malignancy. For example, in glioblastoma, FOSL1 can induce the transformation into the mesenchymal (MES) subtype by indirectly activating NF-κB (33). Moreover, the lack of the neurofibromatosis type 1 gene (NF1) can promote the RAS-MAPK (mitogen-activated protein kinase) pathway and upregulate the expression of FOSL1, which allows the maintenance of the MES subtype characteristics (34). Epithelial–mesenchymal transition (EMT) is also an important step in tumor progression and plays a major role in tumor metastasis and invasion (35). The activity of FOSL1 is necessary for EMT in tumor cells. Transcription factors related to EMT are also known as EMT-TFS, and FOSL1 can act as a mutual regulatory network between them (36). FOSL1 induced by RAS-ERK2 can also directly act on related molecules involved in EMT, such as zinc finger E-box binding homeobox 1 (ZEB1) and zinc finger E-box binding homeobox 2 (ZEB2) (16). In addition, FOSL1 itself is also a metastasis-promoting gene, and the upregulation of its expression promotes cell metastasis (37). Thus, many studies suggest that FOSL1 may be used as a specific indicator of tumors, a target of treatment and a marker of poor prognosis in the future.

However, some studies suggest that FOSL1 may also have an inhibitory effect on the progression of tumors. FOSL1 can induce apoptosis and negatively regulate the activity of AP-1 (3). Therefore, FOSL1 is highly important in the development of tumors, and its relevant regulatory mechanism has attracted increasing attention. Recent studies have shown that there is a mutual regulatory relationship between FOSL1 and ncRNA, and this relationship plays an important role in the occurrence and progression of tumors. For example, in breast cancer, many abnormally expressed ncRNAs (mainly miRNAs and lncRNAs) are associated with the high expression of FOSL1 (38). Studies have shown that FOSL1 can be directly or indirectly regulated as a downstream target (39) and that some ncRNAs can be targeted by its expression (39). However, some studies suggest that FOSL1 can regulate the expression of some ncRNAs, thereby affecting their function in tumors.

## 3 ncRNAs in tumors

**Figure 2:**
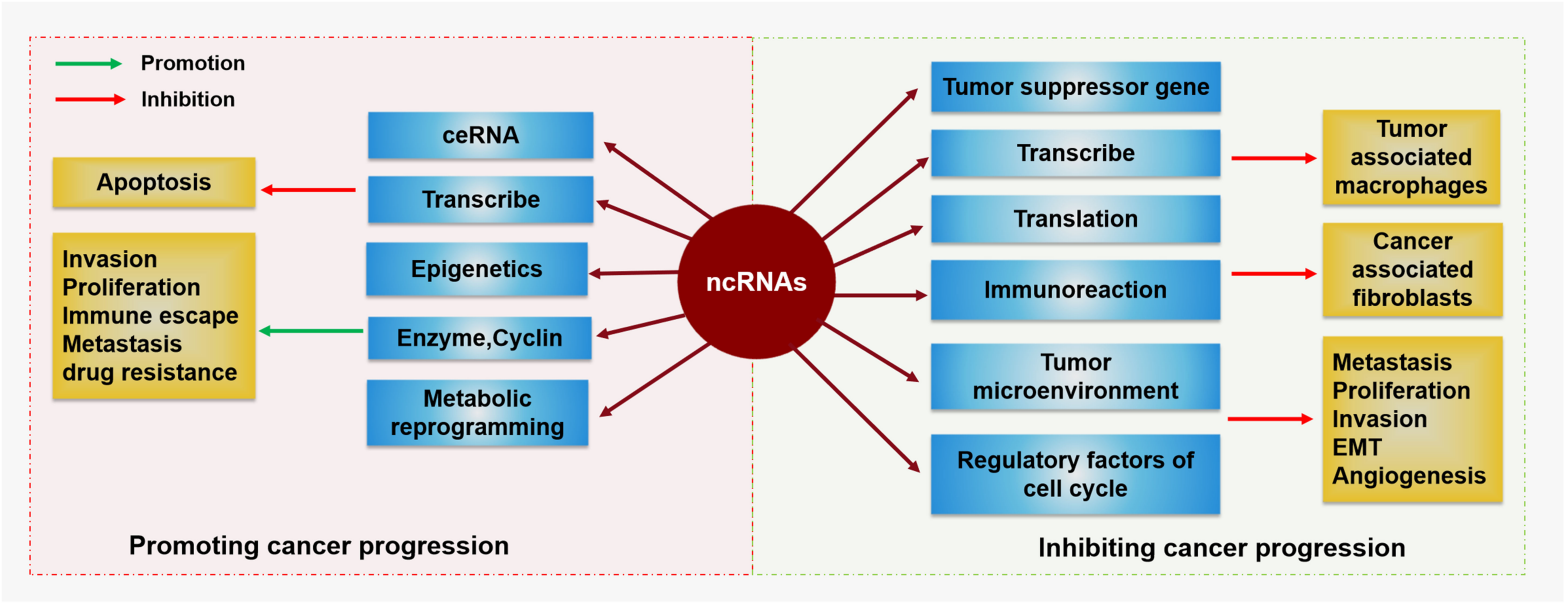
The regulatory function of ncRNAs in cancer ncRNAs are a double-edged sword in the occurrence and development of cancer. On the one hand, ncRNAs can promote tumor cell invasion, proliferation, immune escape, metastasis, or drug resistance by influencing enzymes, cyclins, or metabolic reprogramming but promote apoptosis via ceRNAs, transcription, and epigenetic regulation. On the other hand, ncRNAs can regulate tumor-associated macrophages or cancer-associated fibroblasts by affecting tumor suppressor genes, transcription, translation, or immunoreactions. They can also inhibit tumor metastasis, proliferation, invasion, EMT, or angiogenesis by regulating the tumor microenvironment and regulatory factors of the cell cycle.

Because ncRNAs are abundant in the human genome, they play important roles in the human body and can play regulatory roles in cell proliferation, differentiation, apoptosis and other cell life processes (40). The occurrence of cancer is related to abnormal cell proliferation and apoptosis. ncRNAs, especially miRNAs and lncRNAs, can influence the occurrence and progression of tumors by regulating cellular processes (41). The regulatory roles of miRNAs in many processes in tumor cells are achieved mainly through translation inhibition and mRNA degradation (41), which affect the expression of tumor-related genes. Moreover, tumor progression affects the content of miRNAs, which often form a positive or negative feedback loop to influence tumor progression (41). Different miRNAs have different functions, and according to their different effects on tumors, they can be divided into oncogene miRNAs and tumor inhibitory miRNAs (42, 43), as shown in **Figure 2**. However, overall, more miRNAs play important roles in tumor inhibition by regulating the cell cycle, influencing the immune response and tumor microenvironment. Interestingly, the same miRNA may also play different roles in different kinds of tumors (44). Moreover, miRNAs can not only regulate the tumor itself but also serve as a bridge to help other molecules, including lncRNAs, achieve their biological effects.

lncRNAs can bind to miRNAs by acting as competitive endogenous RNAs (ceRNAs) or RNA sponges to make them inactive (45, 46) and then promote the synthesis of related proteins. Thus, lncRNAs can affect the cellular processes by interacting with DNA and proteins (47) and can also inhibit or promote the development of tumors. In addition, lncRNAs can also bind to a variety of enzymes and regulate their activities. This regulatory mode is widely observed in mitochondria, the endoplasmic reticulum and ribosomes (47). In addition, lncRNAs play a major role in the posttranslational modification of metabolism-related proteins, including phosphorylation, acetylation and ubiquitination (48, 49). Moreover, lncRNAs play important roles at the gene level, which means that lncRNAs can adjust the modifications of transcription factors and subsequently affect the transcription of genes (50–52). Therefore, lncRNAs can activate some carcinogens or participate in carcinogenic signaling pathways, accelerating the progression of tumors, which may function as diagnostic biomarkers or therapeutic targets in cancer (46, 49).

CircRNAs are another type of ncRNA related to miRNAs that can also function as ceRNAs. Ample evidence suggests that, similar to miRNAs and lncRNAs, circRNAs play important roles in transcriptional and posttranscriptional control of gene expression by acting as miRNA sponges (53) and protein scaffolds (54), as well as interacting with RNA-binding proteins (RBPs) (55), thereby influencing tumor progression.

Therefore, ncRNAs (mainly miRNAs, lncRNAs and circRNAs) have a wide influence on the progression of tumors, and their mechanisms of action and downstream targets, including several cancer-related genes, their expression products, and several crucial signaling pathways, are highly variable. Recently, a correlation between the content of ncRNAs and FOSL1 in some tumors was reported (56). Further studies have shown that there is a mutually regulatory relationship between ncRNA and FOSL1. The relationship between miRNA and FOSL1 is the closest (25), and FOSL1 can serve as a direct target for some upstream miRNAs (57) or regulate a few downstream miRNAs (58). lncRNAs and circRNAs mainly indirectly affect the expression of FOSL1 by regulating miRNAs or other molecules (59). In contrast, the expression or function of miRNAs, lncRNAs, and circRNAs can also be directly or indirectly regulated by FOSL1 (25). These mutually regulatory relationships have important impacts on the occurrence and development of tumors, which will be discussed in detail in the following sections.

## 4 The mutual regulatory relationship between FOSL1 and ncRNA and its effect on tumor development

### 4.1 The FOSL1-miRNA system

**Figure 3:**
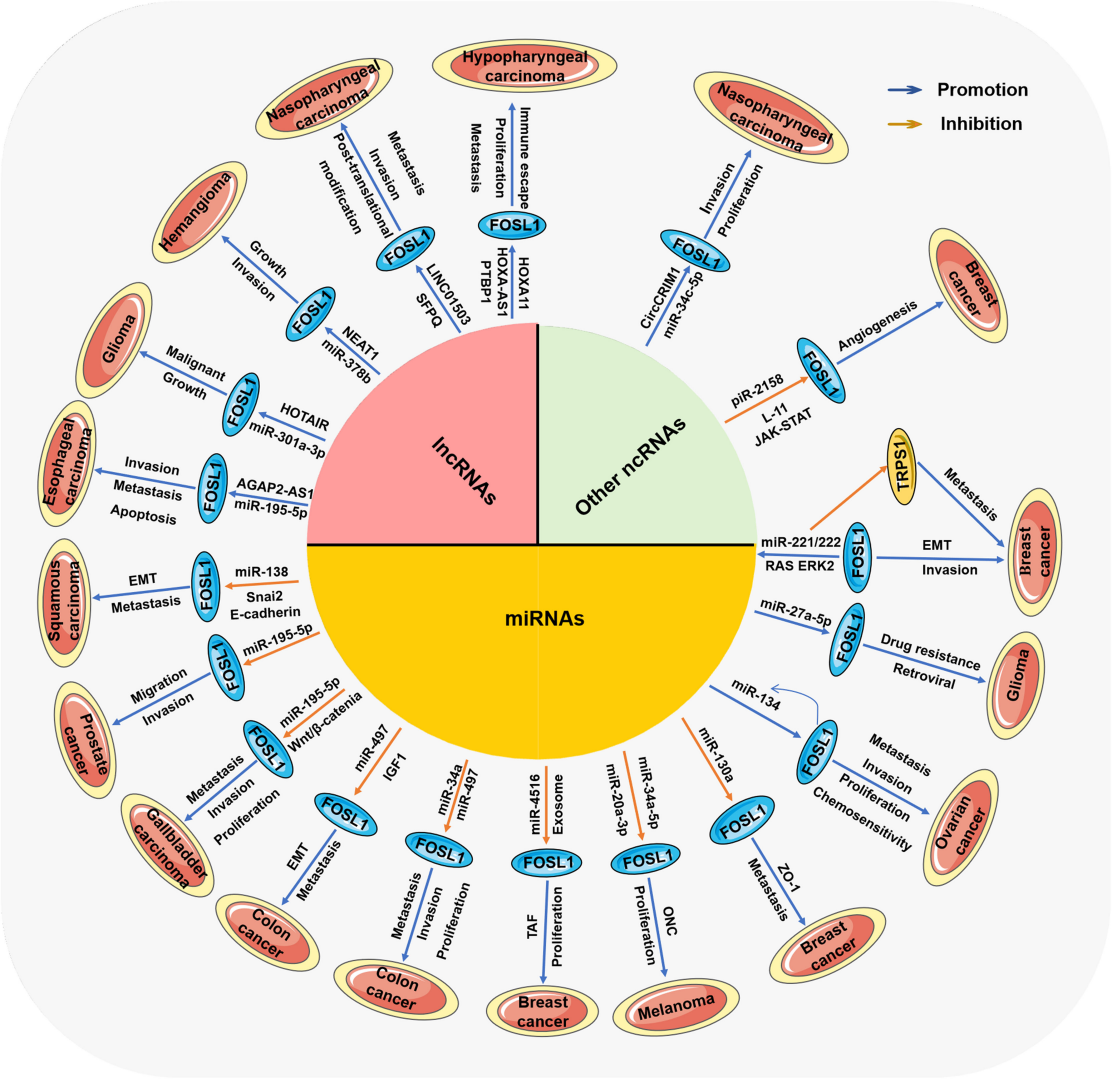
The ncRNA-mediated mechanisms by which FOSL1 affects various tumors Different types of ncRNAs mediate the activation or inhibition of Fra-1/FOSL1 through many signaling pathways, thereby affecting the progression of various tumors.

Among the various molecular mechanisms and relevant molecules involved in the mutual regulation of ncRNAs and FOSL1, miRNAs are the most closely related to FOSL1, as shown in **Table 1** and **Figure 3**. In most cases, miRNAs can inhibit the expression of downstream target genes and reduce the content of specific proteins, whereas FOSL1 can promote transcription. miRNAs can mediate gene silencing by binding their own specific sequence to the specific sequence in the 3’ untranslated region of FOSL1, which then directly regulates the expression of FOSL1 (60). In addition, some miRNAs (for example, miR-138) can also interact with the 5’ *FOSL1* noncoding region and coding region to affect the expression of FOSL1 (25). Moreover, a few miRNAs are regulated by FOSL1 as targeted genes; for example, FOSL1 can affect the transcription of miR-221/222 (61). The relationship between miRNAs and FOSL1 *in vivo* can be divided into two main categories: first, as upstream regulatory molecules, miRNAs can affect FOSL1; second, FOSL1 can also regulate miRNAs and affect the development of tumors. In general, miRNAs act as upstream regulators of FOSL1 and play a role in inhibiting cancers, whereas downstream miRNAs promote the development of tumors.

**Table 1:** Regulation of Fra-1/FOSL1-related ncRNAs in cancer.

ncRNA types	Roles in cancer	Position and function relationship with Fra-1/ FOSL1	Related cellular processes	Related genes or signaling pathways	Tumor types	Refs
miRNAs						
miR-195-5p	Tumor suppressor	Upstream, Inhibition	Metastasis, Invasion	–	Prostate cancer	(62)
miR-195-5p	Tumor suppressor	Upstream, Inhibition	Metastasis, Invasion, Proliferation	Wnt-β-catenia	Gallbladder carcinoma	(66)
miR-138	Tumor suppressor	Upstream, Inhibition	EMT, Metastasis	Snai2, E-cadherin	Squamous cell carcinoma	(57)
miR-497	Tumor suppressor	Upstream, Inhibition	EMT, Metastasis	–	Colon cancer	(69)
miR-34a	Tumor suppressor	Upstream, Inhibition	Metastasis	P53, MMP-1, MMP-9	Colon cancer	(71)
miR-130a	Tumor suppressor	Upstream, Inhibition	Metastasis	ZO-1	Breast cancer	(73)
miR-19a-3p	Tumor suppressor	Upstream, Inhibition	Tumor-associated-macrophage	IL-6, STAT3	Breast cancer	(80)
miR-4516	Tumor suppressor	Upstream, Inhibition	Cancer-associated-fibroblast, Proliferation	Exosome	Breast cancer	(86)
miR-34a-5p, miR-20a-3p	Tumor suppressor	Upstream, Inhibition	Proliferation	ONC	Melanoma	(59)
miR-221/222	Oncogene	Upstream, Promotion	EMT, Invasion	RAS, ERK2, TRPS1	Breast cancer	(89)
miR-27a-5p	Oncogene	Downstream, Promotion	Drug resistance, Retroviral	–	Glioma	(59)
lncRNAs						
AGAP2-AS1	Oncogene	Upstream, Promotion	Invasion, Metastasis, Apoptosis	miR-195-5p	Esophageal carcinoma	(100)
HOTAIR	Oncogene	Upstream, Promotion	Malignant growth	miR-301a-3p	Glioma	(102)
NEAT1	Oncogene	Upstream, Promotion	Growth, Invasion	miR-378b	Hemangioma	(108)
LINC01503	Oncogene	Upstream, Promotion	Metastasis, Invasion, Post-translational modification	SFPQ	Nasopharyngeal carcinoma	(110)
HOXA11-AS1	Oncogene	Upstream, Promotion	Immune escape, Proliferation, Metastasis	HOXA-AS1, PTBP1	Hypopharyngeal carcinoma	(113)
Others						
CircCRIM1	Oncogene	Upstream, Promotion	Invasion, Proliferation	miR-34c-5p	Nasopharyngeal carcinoma	(120)
piR-2158	Tumor suppressor	Downstream, Inhibition	Angiogenesis	IL-11, JAK-STAT	Breast cancer	(124)

-: Not mentioned.

#### 4.1.1 FOSL1 exerts a protumor effect due to the loss of negative regulation by miRNAs

In prostate cancer, the expression of miRNA-195-5p decreases abnormally, and its ability to suppress the expression of FOSL1 decreases, which leads to an increase in the content of FOSL1 and promotes invasion and metastasis of the tumor. Moreover, the upregulation of FOSL1, which is a component of AP-1, can activate AP-1 persistently and participate in the transformation mediated by AP-1 (62, 63). In squamous cell carcinoma, downregulation of miRNA-138 leads to an increase in the expression of FOSL1, which can increase the expression of the transcription inhibitor Snai2. Snai2 can serve as an inhibitor of gene expression and then decrease the expression of E-cadherin, which can promote EMT and the progression of tumors (57, 64, 65). In gallbladder carcinoma, the content of miRNA-195-5p decreases, which increases the expression of its direct target, FOSL1. By upregulating the expression of the transcription factor β-catenin in the Wnt-β-catenin signaling pathway, the function of this signaling pathway is increased, and tumor proliferation, invasion and migration are subsequently promoted (66, 67). In colon cancer, miRNA-497 can inhibit FOSL1, thus reducing the content of FOSL1 and decreasing the content of miR-34a in tumor cells. Moreover, miRNA-497 can also downregulate insulin-like growth factor 1 and inhibit the survival and invasion of cancer cells (68, 69). The expression of miRNAs can also be regulated by some tumor-related genes; for example, miRNA-34a can be a target of the p53 gene (70). Under the action of p53, the expression of miRNA-34a increases, thus inhibiting the expression of FOSL1 and indirectly inhibiting the activity of MMP-1 and MMP-9, which causes the contents of MMP-1 and MMP-9 in colon cancer cells decrease, thus inhibiting the migration and metastasis of tumor cells (71). In addition, many abnormally expressed miRNAs have been found in breast cancer, many of which are involved in mutual regulation with FOSL1 (72). For example, miRNA-130a can reduce the content of FOSL1, and FOSL1 can promote the expression of the tight junction protein Zona occludens 1 (ZO-1) in breast cancer; thus, miRNA-130a can indirectly inhibit ZO-1 through FOSL1 by inhibiting its expression to inhibit tumor metastasis (73, 74). These data indicate that some miRNAs in tumors can play a role in inhibiting cancer by down-regulating the expression or function of FOSL1. When these miRNAs are inhibited, the tumor-promoting effect of FOSL1 will naturally be restored.

Studies have shown that the tumor microenvironment (TME) and tumor-related macrophages have important effects on the occurrence and development of tumors (75–77). In macrophages, the M2 phenotype can promote tumor development (78), while FOSL1 can prevent macrophages from differentiating into the M2 phenotype by activating the STAT3 pathway and other downstream pathways (37). miR-19a-3p can inhibit the expression of FOSL1 at the mRNA level by binding to the 3’UTR, thus indirectly reducing the number of M2 phenotype macrophages and slowing the progression of cancer (79). Moreover, cytokines such as IL-6 can inhibit the expression of miR-19a-3p and promote the development of tumors (80). In addition to the TME, other factors, such as cancer-related fibroblasts (CAFs), may also affect the development of tumors (81). CAFs can secrete vesicles containing various components and abundant miRNAs that are known as exosomes (82). This kind of exosome can promote invasion and drug resistance (83, 84) and can deliver carcinogenic DNA to normal cells (85). In particular, in pancreatic cancer, exosomes play an important role in the resistance of tumor cells to chemotherapy (82). In breast cancer, CAFs can generate exosomes containing miR-4516, which can inhibit the expression of FOSL1, thus indirectly inhibiting the proliferation and invasion of tumor cells (86). Therefore, some drugs, such as Onconase (ONC), can affect the progression of tumors through the downregulation of FOSL1 mediated by miRNAs. ONC, an enzyme that was originally extracted from amphibians and belongs to the secretory ribonuclease superfamily, is cytotoxic and can inhibit the proliferation of tumor cells (87). In melanoma, ONC targets miR-34a-5p and miR-20a-3p and upregulates them, and both of these miRNAs can downregulate the expression of FOSL1 (59). These data suggest that one of these antitumor mechanisms of ONC may involve indirect regulation of specific cancer proteins, such as FOSL1.

#### 4.1.2 FOSL1 exerts a protumor effect by negatively regulating miRNAs

miRNAs can bind to the 3’ noncoding sequence of *FOSL1* to regulate FOSL1, whereas FOSL1 also plays a regulatory role in miRNAs, and this regulation is related to the proto-oncogene that is the upstream regulator of FOSL1, such as IL-6.

RAS can promote the expression of FOSL1 through the downstream positive regulation of ERK2 (88), and FOSL1 can subsequently bind to the promoter region of miR-221/222 to promote its transcription (89). miR-221/222 act on its downstream target, trichorhinophalangeal syndrome type 1 (TRPS1), which is an inhibitor of EMT, and miR-221/222 can reduce its expression, thus promoting the epithelial–mesenchymal transition of tumor cells and increasing tumor invasion (89). Moreover, although FOSL1 may play a dual role in tumor progression, at present, research only supports the carcinogenic effect of FOSL1 in its regulation of miRNAs.

FOSL1 not only affects the occurrence and development of tumors but also affects the drug resistance of tumor cells, and it is beneficial to the survival of drug-addicted cancer cells when the drug is withdrawn (90). miRNAs are also involved in this process. In ovarian cancer, miR-134 can form a positive feedback loop with FOSL1, which reduces the sensitivity of tumor cells to chemotherapy drugs (91). In this loop, miR-134 targets suppressor-of-Dis2-number 2 (SDS22) and inhibits its expression, which indirectly promotes the activation of JNK and ERK and then promotes the expression of FOSL1 and increases the FOSL1 content (92, 93). FOSL1 can bind to the promoter region to promote the transcription of miR-134, forming a feedback loop. Moreover, miR-134 can promote the repair of DNA by promoting the phosphorylation of H2AX S139, thus preventing the influence of chemotherapeutic drugs (94, 95). In glioma, FOSL1 can promote the transcription of miR-27a-5p and miR-221-3p by binding to the promoter region of genes, thus reducing the sensitivity of cancer cells to radiotherapy (58). These data suggest that the antitumor effects of some miRNAs may be lost or become less efficient due to the negative regulation exerted by FOSL1.

### 4.2 The FOSL1-lncRNA system

Compared with the complex relationship between miRNAs and FOSL1, the regulatory effect of lncRNAs on the expression of FOSL1 is simpler, as shown in **Table 1** and **Figure 3**. lncRNAs are usually upstream regulatory molecules that can indirectly adjust the expression of FOSL1 by regulating the expression of miRNAs and other molecules. First, lncRNAs act as “RNA sponges”, which reduce the content of miRNAs and weaken their function through adsorbing miRNAs (96, 97), thus alleviating the inhibitory effect of miRNAs on the expression of FOSL1 and indirectly promoting the expression of FOSL1. In addition, lncRNAs can also regulate the activities of some enzymes related to transcription and RNA modification (98, 99), thus affecting the transcription and translation of FOSL1 and regulating FOSL1. In this process, lncRNAs usually promote the progression of tumors, which is beneficial for the growth, proliferation, invasion and metastasis of tumor cells. However, to date, lncRNAs have not been shown to be directly regulated by FOSL1.

#### 4.2.1 The tumor-promoting effect of FOSL1 is positively regulated by lncRNAs that target miRNAs

In esophageal carcinoma, the expression of the lncRNA AGAP2-AS1 increases, and AGAP2-AS1 can bind to miR-195-5p, resulting in a reduction in the content of FOSL1, a target gene of miR-195-5p, which then promotes the invasion and metastasis of cancer or inhibits apoptosis (100). In glioma, the oncogenic lncRNA HOTAIR can promote the occurrence and metastasis of tumors (101) and is highly expressed in glioma (102), colorectal cancer (103) and lung cancer (104). HOTAIR plays an important role in tumor growth, especially in glioma. HOTAIR can bind to miR-301a-3p as a ceRNA to inhibit its negative regulation of FOSL1 and then promote the expression of FOSL1 (105). Moreover, as an important regulator in glioblastoma, the activity of the lncRNA TRPM7 is related to the malignant growth of tumors (106). Transient receptor potential melastatin 7 (TRPM7) can promote the expression of HOTAIR and then indirectly regulate the progression of cancer as a negative regulator of miR-301a-3p (105). The expression of the lncRNA NEAT1, which can adsorb miR-378b by acting as an RNA sponge, is abnormal in hemangioma; moreover, FOSL1 was shown to be a downstream target gene of miR-378b, and its expression was inhibited by miR-378b (107). Therefore, NEAT1 can indirectly promote the expression of FOSL1 by adsorbing miR-378b. Interestingly, NEAT1 is also regulated by other molecules to further regulate the miR-378b-FOSL1 axis. For example, alkyl repair homologous protein 5 (ALKBH5) can reduce the m^6^A modification of NEAT1 (108, 109), thus promoting the expression of NEAT1 and its downstream molecular mechanism at the transcriptional level. In other words, the ALKBH5-NEAT1-miR-378b-FOSL1 axis plays important roles in the development of cancer.

#### 4.2.2 The tumor-promoting effect of FOSL1 is positively regulated by lncRNAs that target other molecules

In nasopharyngeal carcinoma, the lncRNA LINC01503 can play a regulatory role in FOSL1 by influencing the SFPQ-FOSL1 axis (110). LINC01503 can recruit the splicing factor SFPQ (splicing factor proline/glutamine-rich), which can promote the posttranscriptional modification of FOSL1 and indirectly increase the expression of FOSL1 (111, 112). In hypopharyngeal carcinoma, the lncRNA HOXA11-AS1-polypyrimidine tract-binding protein 1 (PTBP1)-FOSL1-programmed death ligand 1 (PD-L1) axis has been identified as a new pathway that affects the immune escape of tumors from T cells and influences the proliferation and metastasis of tumors by regulating PD-L1 (111, 112). In this pathway, HOXA11-AS1 can increase the content of PTBP1, which can increase the content of FOSL1 by increasing the stability of the FOSL1 mRNA (113, 114), subsequently promoting the expression of PD-L1, which is beneficial for the proliferation and immune escape of cells. These results indicate that in addition to negatively regulating tumor-suppressive miRNAs, lncRNAs can also positively regulate the protumor effect of FOSL1 by targeting molecules upstream of FOSL1.

### 4.3 Systems involving FOSL1 and other ncRNAs

In addition to miRNAs and lncRNAs, other types of ncRNAs affect tumors, such as circRNAs and piRNAs (115), as shown in **Table 1** and **Figure 3**. They can also regulate various molecular mechanisms and participate in the occurrence and development of tumors. Among these other types of RNA, circRNAs and piRNAs are currently reported to have regulatory relationships with FOSL1. Both circRNAs and piRNAs can regulate FOSL1. CircRNAs are closed circular RNA molecules that are highly stable, and their expression is specific to different tissues (116). CircRNAs also play a role in cellular processes such as gene expression and protein synthesis (117–119). The content of CircCRIM1 is increased in nasopharyngeal carcinoma and affects the proliferation and invasion of cancer cells. This effect may be related to the regulation of the miR-34c-5p-FOSL1 axis by CircCRIM1 (120). In tumor cells, CircCRIM1 can bind to miR-34c-5p to prevent its function and then indirectly promote the expression of FOSL1. These studies suggest that, similar to lncRNAs, circRNAs can also indirectly affect FOSL1, mainly by regulating other molecules, including miRNAs.

piRNAs are small noncoding RNAs with a length of approximately 30 nt that were first discovered in microbes (121). PiRNAs can bind to the PIWI protein to form a piRNA-induced silencing complex, which plays a role in gene expression and epigenetic regulation (122). Angiogenesis is necessary for the growth of tumors and distant metastasis in patients with breast cancer (123). FOSL1 can increase this process, whereas the overexpression of piR-2158n can inhibit this phenomenon (124). FOSL1 can bind to the promoter region of interleukin-11 (IL-11) and promote transcription (125). The overexpression of IL-11 can further activate the downstream Janus kinase-activator of transcription (JAK-STAT) signaling pathway, subsequently promoting the development of cancer (126). However, piR-2158 can compete with FOSL1 for the binding site on the IL-11 promoter region, which makes the function of FOSL1 ineffective and plays an anticancer role. In other words, unlike circRNAs, piRNAs act as competitive inhibitors of FOSL1 by competing for the binding site on the downstream target, thus blocking the functional pathway of FOSL1.

## 5 The FOSL1-ncRNA system may serve as a potential cancer treatment strategy

At present, many studies have focused on the role of ncRNAs and FOSL1 in cancer and their clinical significance. Among all the ncRNAs, miRNAs and lncRNAs are highly expressed *in vivo*, which has attracted increasing attention. Abnormal expression of miRNAs and lncRNAs is often found in tumors, and their expression is specific to different types of cancer (127). Therefore, the type of cancer can be identified at an earlier stage by assessing the contents of miRNAs and lncRNAs in the tumor cells. Treatments that target different kinds of miRNAs also vary depending on their categories and effects. Carcinogenic ncRNAs are overexpressed in tumors and can promote the development of cancer, and therefore, it is necessary to inhibit these miRNAs to control the tumors. At present, therapies mainly include anti-ncRNA oligonucleotides, ncRNA sponges and small-molecule inhibitors (128). The main function of the first two types of therapy is to bind to or absorb the miRNAs, thus blocking their function. The scope of action of small-molecule inhibitors is wider and includes affecting biosynthesis, target-effect binding and many other aspects (90). However, tumor suppressor miRNAs are often downregulated in tumors, and therefore, increasing their content to inhibit the development of cancer is the main strategy. miRNA mimics and miRNA replacement therapy have been shown to increase the content of ncRNA in a model (128). Moreover, therapies targeting FOSL1 involve multiple pathways, including genes, mRNAs, protein degradation and small-molecule inhibitors (129). Initial results on innovative vectors and the application of CRISPR (clustered regularly interspaced short palindromic repeats)-Cas9 (CRISPR-associated nuclease 9) based on the CRISPR system have been reported (90). The stability of the protein mainly depends on the C-terminal sequence, which means that the degradation of FOSL1 can be induced by a protein degradation agent (90). In addition to RNAi, miRNAs can also bind to mRNAs to inhibit the translation of proteins (41). In summary, the diagnostic capabilities and potential targeted therapeutic strategies of ncRNAs and FOSL1 in tumors are increasingly being explored.

Interestingly, the relationship between ncRNAs and FOSL1 has recently attracted attention for its clinical application. The mutual regulation of ncRNAs and FOSL1 can affect the development of cancer, and therefore, researchers hope to use them as new targets in cancer treatment. In glioma, FOSL1 can reduce the sensitivity of tumor cells to radiotherapy by inhibiting miR-27a-5p, and therefore, FOSL1 silencing can improve the curative effect of radiotherapy (58). miR-195-5p can inhibit FOSL1 in prostate cancer, thus inhibiting tumor migration and invasion. miR-195-5p has been suggested as a new therapeutic target for prostate cancer (62). In breast cancer, miR-4516 in exosomes can inhibit the progression of FOSL1-dependent breast cancer, and some studies suggest that miR-4516 may become a new anticancer drug for therapy. The combination of miRNA-related therapy and chemotherapy may become a new strategy for the treatment of breast cancer (86). The feedback loop of FOSL1-miR-134-SDS22 plays an adverse role in the response of ovarian cancer to chemotherapy, and such a treatment may improve the effect of chemotherapy in ovarian cancer (91). Similarly, some lncRNAs in the FOSL1-ncRNA system can also be therapeutic targets. For example, HOTAIR can promote the development of cancer through the HOTAIR-miR301a-3p-FOSL1 axis, suggesting the possibility of finding new therapeutic targets and biomarkers (105). Silencing AGAP2-AS1 can indirectly inhibit FOSL1 and can thus inhibit tumor metastasis, which is expected to be a targeted therapeutic strategy in the future (100). ALKBH5 can promote the development of tumors through the NEAT1-miR-378b-FOSL1 axis, and therapy against these molecules may become a new method for treating infantile hemangioma (IH) (107). In addition, inhibitors targeting the LINC01503-SFPQ-FOSL1 axis in nasopharyngeal carcinoma may become new targeted therapies (110). Moreover, other ncRNAs of the FOSL1-ncRNA system are expected to become new targets for tumor treatment. For example, piRNA-2158 can inhibit the promoting effect of FOSL1 by acting on IL-11 and the formation of new blood vessels in tumors, which provides new ideas for the treatment of breast cancer (124). These studies indicate that the FOSL1-ncRNA system may be a potential target for tumor therapy, although there are currently no reports of preclinical/clinical trials targeting this axis.

## 6 Conclusion and discussion

The main functions of FOSL1 in tumor development have been confirmed, including promoting angiogenesis, increasing the heterogeneity of tumor cells, and promoting EMT and tumor invasion and metastasis, and these effects have been found in many tumors. Moreover, FOSL1 is related to various signaling pathways and molecular mechanisms, which can activate various targets and exert various biological effects. FOSL1 also has a variety of regulatory mechanisms, and its expression and regulation are related to the RAS gene and p53 gene. In addition to cancer-related genes, the expression of FOSL1 is also regulated by ncRNAs, with miRNAs promoting the degradation of mRNAs by binding to the 3’ noncoding region and downregulating the expression of FOSL1 at the mRNA level, whereas lncRNAs, circRNAs and piRNAs indirectly affect the expression of FOSL1 by regulating the expression or activity of miRNAs or other related molecules; at the same time, some ncRNAs can also be regulated by FOSL1 as direct downstream targets.

Some cancer-related ncRNAs are transcribed from cancer-related genes, and their expression and molecular activity are often regulated by genes. Moreover, the expression of these genes can also be regulated by ncRNAs. The abnormal expression of FOSL1 in tumor tissues is often related to oncogenes, and some oncogenes can indirectly affect the expression of specific miRNAs by regulating FOSL1, thus affecting the progression of tumors. In addition, FOSL1 can also change the tumor microenvironment by activating downstream signaling pathways and affect the immune response of the body’s immune system against tumors. There is a mutual regulatory relationship between the above molecules, which together constitute a regulatory network related to FOSL1 in tumors and the tumor microenvironment. This regulatory network includes cancer-related genes, ncRNAs, related transcription factors and regulatory factors, FOSL1 and its downstream pathways, tumor tissues and the tumor microenvironment, which have important impacts on the occurrence and progression of tumors. This network involves multiple molecular pathways, and the mutual regulation of each part involves a variety of molecules and genes. However, the effect of the relationship between FOSL1 and ncRNA on tumors is not completely clear and may become one of the directions of tumor research in the future. Moreover, targeted molecular therapy for tumors is related mainly to the specificity of targeted molecules and the inherent sensitivity of tumor cells and their microenvironment. However, different types of tumors show different sensitivities or tolerances to different molecular treatment methods, which leads to different treatment results. Therefore, identifying the role of the FOSL1-ncRNA axis in tumor activities is very important and requires many laboratory and preclinical studies. Additional relevant ncRNAs in the FOSL1-ncRNA system with diagnostic and therapeutic value are expected to be discovered, and more interaction mechanisms are expected to be revealed.

## Glossary

ALKBH5: Alkyl repair homologous protein 5
AP-1: Activator protein
ATF: Activating transcription factor
bZIP: Basic leucine zipper
CAF: Cancer-related fibroblast
ceRNA: Competitive endogenous RNA
circRNA: Circular RNA
CRISPR: Clustered regularly interspaced short palindromic repeats
Cas9: CRISPR-associated nuclease 9
CSCs: Tumor stem cells
DCDK4: Cyclin-dependent kinase 4
EMT: Epithelial–mesenchymal transition
EMT-TFs: Transcription factors related to EMT
Fra-1: Fos-related antigen 1
IL-6/11: Interleukin-6/11
JAK: Janus kinase
lncRNA: Long noncoding RNA
MAF: Muscle aponeurotic fibrosarcoma
MES: Mesenchymal
miRNA: MicroRNA
MMP-1/9: Matrix metalloproteinase 1/9
ncRNAs: noncoding RNAs
NF1: Type 1 gene
ONC: Onconase
PD-L1: Programmed death ligand 1
PI3K: Phosphatidylinositol 3-kinase
piRNA: Piwi-interacting RNA
PTBP1: Polypyrimidine tract-binding protein 1
ERK: Extracellular signal-regulated kinase
MAPK: Mitogen activated protein kinase
RB: Retinoblastoma protein
RBPs: RNA-binding proteins
SDS22: Suppressor-of-Dis2-number 2
SEs: Super enhancers
SFPQ: Splicing factor proline/glutamine-rich
STAT: Activator of transcription
Stat3: Signal transducer and activator of transcription 3
TME: Tumor microenvironment
TPA: Tissue plasminogen activator
TREs: TPA response elements
TRPM7: Transient receptor potential melastatin 7
TRPS1: Trichorhinophalangeal syndrome type 1
ZEB1/2: Zinc finger E-box binding homeobox 1/2
ZO-1: Zona occludens 1.
